# Community pharmacist interventions in ear health: a scoping review

**DOI:** 10.1017/S1463423621000487

**Published:** 2021-11-03

**Authors:** Selina Taylor, Alice Cairns, Shaun Solomon, Beverley Glass

**Affiliations:** 1 Centre for Rural & Remote Health, James Cook University, Mount Isa, Australia; 2 College of Medicine and Dentistry, James Cook University, Townsville, Australia

**Keywords:** ear health, expanded pharmacy practice, Indigenous ear health, models of care pharmacy practice, scope of practice

## Abstract

**Background:**

In Australia, around 3.6 million people suffer from hearing loss, more than 1.3 million with preventable hearing conditions. Ear diseases are prevalent in Indigenous populations, particularly children and are associated with poor educational outcomes and subsequent high rates of unemployment and incarceration. In Australia, rural and remote communities have rates of middle ear perforations five times the rate that the World Health Organisation regards to be a significant public health problem.

Barriers to accessing ear health services have been identified including gaps in testing during the ‘early years’ and difficulty in accessing these services. Reducing the risk of hearing loss through improved ear health care can directly impact the ability to learn and develop. Collaboration between community, health providers and government is crucial to ensure necessary support for change. An opportunity presents for rural community pharmacists, who are both qualified and accessible to provide an ear health programme and thus improve health outcomes for both Indigenous and non-Indigenous Australians in their communities.

**Aim:**

The aim of this study was to identify published evidence of pharmacists’ involvement in ear health care interventions to inform the development of ear health services able to be delivered in rural community pharmacy in Australia.

**Data sources:**

The search strategy was applied to the following electronic databases: MEDLINE, Scopus, CINAHL, Emcare, Cochrane, Google Scholar and Google.

Study selection articles were included if they described an ear health intervention in a community pharmacy setting. The interventions reported in the articles were evaluated for their inclusion of effectiveness, whether the service was sustainable, and the inclusion of enablers and barriers to the provision of ear care. The articles were also thematically analysed using the *Deadly Ears Deadly Kids Deadly Communities Framework*. A total 8427 articles were identified and evaluated against inclusion and exclusion criteria, with eleven eligible articles suitable for inclusion in the review. The articles included were conducted in Australia (*n* = 4), England (*n* = 4), United States of America (*n* = 2) and Brazil (*n* = 1). The ear health interventions identified included hearing screening (*n =* 3), otoscopy pilot studies (*n =* 2), audiometry services (*n =* 1), specific education for undergraduate pharmacy students (*n =* 2) and a pharmacy-based clinic (*n =* 3). Effectiveness and sustainability were not formally reported in any of the included articles. Positive outcomes, funding availability, consumer access to community pharmacy, cost savings for consumers and improved connection to health providers were identified as enablers. Difficulty in attracting funding was the most commonly reported barrier.

**Conclusions:**

Improving ear health of both Indigenous and non-Indigenous peoples through services provided in community pharmacy presents as an important opportunity for rural pharmacists. Pharmacists are accessible and thus well placed to improve ear healthcare and resultant quality of life for these vulnerable populations. This review has identified factors required to effectively develop ear health models of care in community pharmacy including a pharmacist training program, continuous funding to ensure sustainability and support from pharmacy stakeholders and the community.

## Introduction

Ear healthcare is a complex and multifaceted area of health with significant gaps in prevalence data and burden of disease outcomes (Macquarie University, 2019). Indigenous populations, in particular, experience high rates of ear disease with strong links to social determinants of health, presenting significant challenges for managing ear disease (Macquarie University, 2019). Globally, Indigenous populations are at a high risk of hearing loss with an estimated 6.1% of the world’s population living with hearing loss and an annual cost of unaddressed hearing loss of 750 billion USD (Macquarie University, 2019; World Health Organisation, 2020). Ear problems are increasing with the World Health Organisation (WHO) proposing that by 2050, if no change is made, the global number of people with disabling hearing loss would reach 900 million, double that of 2019 (Macquarie University, 2019). These global numbers have been progressively increasing with 90% of the burden of hearing loss representing low- and middle-income countries (LMI) (Macquarie University, 2019). In Australia, it is Indigenous Australians who experience the greatest burden of disadvantage and disability due to hearing problems (Macquarie University, 2019).

A similar situation of hearing loss is occurring in Australia, with around 3.6 million people suffering from hearing loss and more than 1.3 million diagnosed with preventable hearing conditions (Australian Government Department of Health, 2020). The Australian healthcare system is recognised as the second best in the world, yet Australia still has some of the highest rates of chronic middle ear disease in Indigenous children (Macquarie University, 2019). Middle-ear disease in Australian Indigenous peoples is considered by the WHO to be a ‘massive public health problem’, double the prevalence of what is deemed an emergency public health situation. Australian Indigenous children experience otitis media (OM) at a younger age at first episode, with a higher frequency of infection, with greater severity and greater persistence than non-Indigenous children (Australian Institute of Health and Welfare, 2014). In some remote communities in Northern and Central Australia, the prevalence of middle-ear disease or OM is as high as 50% in children under three years of age. The consequences of ear disease are poorer educational, social and behavioural outcomes and disrupted connection to land, culture and community, which can result in more frequent contact with the criminal justice system (Macquarie University, 2019).

Inadequately trained human resources and a lack of infrastructure and supplies have been described as key challenges to the provision of ear and hearing care internationally (Macquarie University, 2019). First nations people often have limited access to health care, which can result in delayed diagnosis, treatment and management of middle ear disease, which contribute to prolonged periods of hearing loss and impairment (Australian Institute of Health and Welfare, 2018). In addition, Australia currently experiences shortages of rural and remote health care workers able to provide ear health and this is predicted to worsen in the future (Hearing Health Sector Committee, 2019). In 2016, a book was published on ‘Hearing Health Care of Adults: Priorities for Improving Access and Affordability’. This title describes hearing health services and has a section describing the professionals involved in hearing healthcare. Audiologists, hearing instrument specialists, otolaryngologists and primary care providers described as GPs and nurses are included, though there is no description for Indigenous Health Workers or Pharmacists. This highlights the limited consideration for other health providers, who may have better access to communities, including community pharmacists who are permanent members in rural communities, to be considered as health professionals involved in ear healthcare (Committee on Accessible and Affordable Hearing Health Care for Adults, 2016).

Urgent work is needed to develop sustainable and accessible models of care in rural and remote Australia to address this issue (Australian Institute of Health and Welfare, 2018; Macquarie University, 2019). Of importance is the need to ensure that health care is provided in a way that embraces culturally responsive care, which is an extension of patient-centred care that includes focused attention on social and cultural factors (Pharmaceutical Society of Australia, 2014). Community pharmacists may offer an opportunity to address some of this need. Globally, there are examples of pharmacists working in expanded roles to better address health needs by the provision of services in addition to traditional medicine supply roles (Hoti *et al.*, 2011). In rural and remote areas, pharmacists are trusted and accessible health professionals, who are trialling expanded services to meet local community needs (Taylor *et al.*, 2019). Therefore, community pharmacists as potential contributors to close the gap on ear disease is an innovative strategy to consider for Australian communities.

This scoping review aims to identify published evidence of pharmacists’ involvement in ear health care interventions to inform the design and development of ear health services to be delivered in rural community pharmacy in Australia.

## Methods

### Protocol

**Figure 1.**
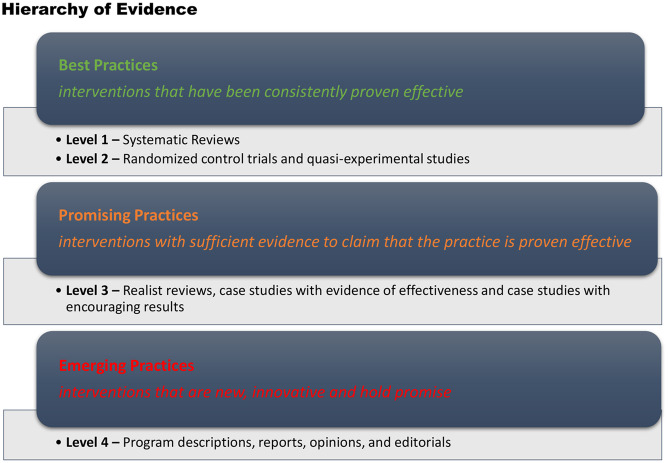
Canadian hierarchy of promising practices evidence (Canadian Homelessness Research Network, 2013)

A scoping review was conducted using the Arksey and O’Malley’s methodological framework (Arksey & O’Malley, 2005; Moher *et al.*, 2009; Levac *et al.*, 2010). The review was conducted in accordance with the Preferred Reporting Items for Systematic reviews and Meta-Analyses (PRISMA-ScR) extension for scoping reviews guideline (Moher *et al.*, 2009; Tricco *et al.*, 2018) (Figure 1). An initial review of the Cochrane database for systematic reviews and other databases undertaken to determine whether reviews existed or were in progress did not identify reviews on ear health intervention by pharmacists.

The purpose of this scoping review is to identify published evidence of pharmacists’ involvement in ear health care interventions to inform the development of ear health services to be delivered in rural community pharmacy in Australia. In particular, the review aimed to explore effectiveness (are there positive outcomes?), sustainability (can the model be continued long term?) and enablers and barriers to these pharmacy-based ear health interventions.

### Search strategy

An initial search of MEDLINE and Scopus was conducted to identify key words found in the title, abstract and index terms appropriate for the search strategy. Keywords identified were (pharmac* OR “Drug Store” OR “Drug Stores” OR Drugstore* OR apothecar* OR chemist) AND (ear OR ears OR “vestibulocochlear apparatus” OR “vestibulocochlear system” OR hearing OR hypoacuses OR hypoacusis OR deafness). A second search using all identified keywords and index terms was then conducted on 18 May 2020 in the databases MEDLINE, CINAHL, EmCare, Informit and Scopus. In addition, the first 20 pages of Google and Google Scholar keyword searches were included. The reference lists of all identified records and articles were also searched for additional articles and resulted in none being found.

Study participants included pharmacy undergraduate students, intern pharmacists, registered pharmacists, pharmacy assistants and consumers. For articles to be included in the scoping review, there needed to be a clear connection between an ear health intervention and pharmacy. The context of the articles included was university curriculum, community and retail pharmacies. Articles reporting services provided by hospital pharmacies were excluded.

### Eligibility criteria

All published data on ear heath interventions provided in pharmacies with no date or language restriction were included. As literature on this topic is limited, no restrictions were placed in regard to study design, and as a result, published news articles, narrative articles and editorials were also included.

Inclusion and exclusion criteria followed the PICOT structure and is reported in Table 1.

**Table 1.** Summary of articles included in review

Reference	Country	Intervention Type/Description	No. Participants	Study Design	Outcomes [X] in box indicates if study described effectiveness, sustainability, enablers and barriers	Level of Evidence/Key Priority Area
Duncan, Clark and Wang, 2016	United States of America	Pharmacy-based clinic Examination of costs and utilization of people <20 years receiving care for AOM in retail pharmacy clinics.	5101 episodes of care for acute otitis media (AOM)	Retrospective cohort study	Pharmacy-based retail clinic visits for AOM cost USD $30–130 less than community pharmacy visits. Antibiotic prescription fill rate was higher for retail clinic episodes (95.4%) than other provider community episodes (82.8%). □ Effectiveness ⊠ Sustainability □ Enablers □ Barriers	Level 3 – Promising Practice
						Key Priority Areas – 3 – Treatment and Support
Pisano and Miller, 2018	United States of America	Undergraduate pharmacy student training Online program to train students to counsel patients with hearing loss for improved medication adherence and understanding.	92 students	Cross-sectional descriptive study	80% agreed the module improved understanding of psychological consequences of hearing loss and ability to counsel patients with hearing loss. ⊠ Effectiveness □ Sustainability □ Enablers □ Barriers	Level 3 – Promising Practice
						Key Priority Areas – 3 – Treatment and Support 5 – Workforce Development
Souza Anacleto de Araújo *et al.*, 2019	Brazil	Undergraduate pharmacy student training Examination of sign language courses available in pharmacy schools in Brazil.	35 pharmacy schools	Cross-sectional descriptive study	18 of 35 pharmacy schools included a sign language course in their curriculum, all of which were elective courses. ⊠ Effectiveness □ Sustainability ⊠ Enablers ⊠ Barriers	Level 3 – Promising Practice
						Key Priority Areas – 3 – Treatment and Support 5 – Workforce Development
Postscript Magazine, 2016	Australia	Hearing screening service Opinion piece targeted at pharmacy assistants to increase knowledge of hearing loss.	–	Editorial – Professional Practice Journal	Highlights the opportunity for pharmacy assistants to identify and refer patients to a hearing specialist. □ Effectiveness □ Sustainability ⊠ Enablers □ Barriers	Level 4 – Emerging Practice
						Key Priority Areas – 2 – Screening, Surveillance and Diagnosis 4 – Partnerships
Lakhani and Eynon-Lewis, 2009	England	Pharmacy-based clinic Opinion piece related to pharmacy management of ear complaints.	–	Editorial – Opinion article	Described lack of support for pharmacists to provide ear health service due to no formal training in the condition. □ Effectiveness □ Sustainability ⊠ Enablers ⊠ Barriers	Level 4 – Emerging Practice
						Key Priority Areas – 3 – Treatment and Support 5 – Workforce Development
The Australian Journal of Pharmacy, (2014)	Australia	Hearing screening service News article describing a hearing screening service provided in community pharmacy by partnership with external hearing services.	>000 participants	Editorial – Professional Practice Journal	Free hearing checks provided in community pharmacies by external provider. □ Effectiveness □ Sustainability ⊠ Enablers □ Barriers	Level 4 – Emerging Practice
						Key Priority Areas – 2 – Screening, Surveillance and Diagnosis 4 – Partnerships
Australian Pharmacist, 2016	Australia	Hearing screening service News article describing hearing screening service provided in community pharmacy by partnership with external hearing services.	–	Editorial – Professional Practice Journal	Free hearing checks provided in community pharmacies by external provider with referral for full hearing assessment if required. Duration 15min. □ Effectiveness □ Sustainability ⊠ Enablers □ Barriers	Level 4 – Emerging Practice
						Key Priority Areas – 2 – Screening, Surveillance and Diagnosis 4 – Partnerships
Warnbro Pharmacy, 2020	Australia	Audiology service Community pharmacy webpage describing audiology service provided in community pharmacy by partnership with external hearing services.	–	Editorial – Service advertisement	Free service including otoscopic inspection, air conduction tone test and if required referral for full hearing assessment. Duration 15–30mins. □ Effectiveness □ Sustainability ⊠ Enablers □ Barriers	Level 4 – Emerging Practice
						Key Priority Areas – 2 – Screening, Surveillance and Diagnosis 3 – Treatment and Support 4 – Partnerships
Independent Community Pharmacist, 2016 *Note this article describes the same intervention as reference (Weinbren, 2016)	England	Otoscopy pilot service News article describing community pharmacy service whereby pharmacists use otoscopy to improve ear health management.	–	Editorial – Professional Practice Journal	Description of a six-month pilot program with pharmacists trained by specialist nurses to examine an ear with an otoscope, diagnose and treat appropriately. □ Effectiveness ⊠ Sustainability ⊠ Enablers □ Barriers	Level 4 – Emerging Practice
						Key Priority Areas – 2 – Screening, Surveillance and Diagnosis 3 – Treatment and Support 5 – Workforce Development
Hall *et al.*, 2019	England	Pharmacy-based clinic Pharmacy service provided by trained pharmacists to provide self-care strategies, supply of non-prescription medicines, or specified prescriptions only medicines, including antibiotics.	408 patients (32% diagnosed with otitis media)	Cohort study	Of 408 patients, 32% were diagnosed with acute otitis media. Overall 309/408 patients were followed up, 85% had not seen another health professional. 96% participants were satisfied with the service. ⊠ Effectiveness ⊠ Sustainability ⊠ Enablers ⊠ Barriers	Level 3 – Promising Practice
						Key Priority Areas – 2 – Screening, Surveillance and Diagnosis 3 – Treatment and Support 5 – Workforce Development
Weinbren, 2016 *Note this study describes the same intervention as reference (Independent Community Pharmacist, 2016)	England	Otoscopy pilot service News article describing community pharmacy service whereby pharmacists use otoscopy to improve ear health management.	–	Editorial – Professional Practice Journal	Six-month otoscopy pilot with positive feedback and potential savings to general practitioner time. ⊠ Effectiveness ⊠ Sustainability ⊠ Enablers ⊠ Barriers	Level 4 – Emerging Practices
						Key Priority Areas – 2 – Screening, Surveillance and Diagnosis 3 – Treatment and Support 5 – Workforce Development

### Data extraction and quality assessment

**Figure 2.**
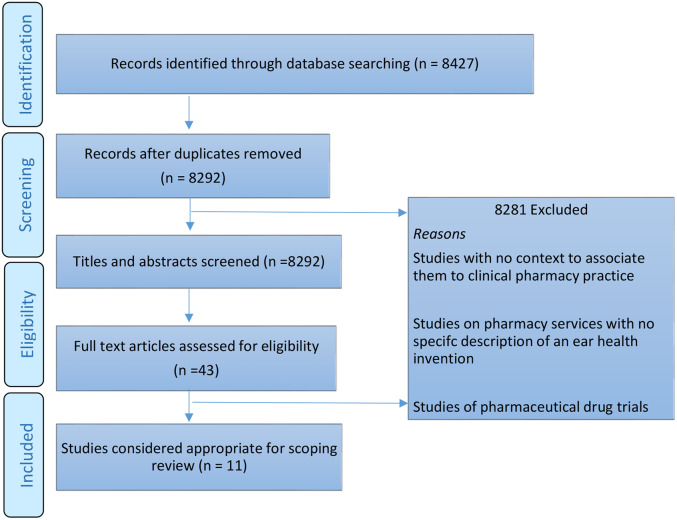
*PRISMA Flow diagram for systematic review* (Moher *et al.*, 2009, Tricco *et al.*, 2018)

Two authors reviewed the search results and applied two quality screens to the included articles, which varied in study design. Discrepancies were resolved by consensus. Hong and Pluye (2018) provide a conceptual framework that considers three components; methodological quality, conceptual quality and reporting quality (Hong & Pluye, 2018). Although articles were examined using this framework, due to the emerging nature of this topic, all articles were included regardless of their methodological rigour, as they all provided some valuable insight into the topic. The second quality screen was conducted using the Canadian Hierarchy of Promising Practices Evidence (Canadian Homelessness Research Network, 2013). This hierarchy provides four levels of evidence, in three categories ranging from best practice through to emerging practices (Canadian Homelessness Research Network, 2013) (Figure 2).

Data were extracted and thematically analysed against the *Deadly Ears Deadly Kids Deadly Communities Framework* (Durham *et al.*, 2015). This framework aims to reduce the incidence and impact of conductive hearing loss associated with OM by intervening at local and national levels and across different sectors. Although articles included in this scoping review were not exclusively targeting OM, the structure of this framework can be applied to ear health broadly, as it has been in this review. There are six key action areas included in the framework:

- Key Action Area 1 – Prevention
- Key Action Area 2 – Screening, Surveillance and Diagnosis
- Key Action Area 3 – Treatment and Support
- Key Action Area 4 – Partnerships
- Key Action Area 5 – Workforce Development
- Key Action Area 6 – Information and Knowledge

These key action areas have been developed to describe the various areas of health care that need to be addressed to improve ear health. They can be applied to the areas in which ear health interventions may be situated and thus provide a suitable framework for the thematic analysis. These key action areas formed the initial theme list, and data extracted from the articles were analysed using an inductive and deductive approach. All data extracted could be applied to these action areas and no additional themes were found. Two researchers analysed the data to minimise objectivity, assumed knowledge and bias. Discrepancies were resolved by consensus.

## Results

### Search results

A total of 8427 articles were identified through online database searching (Emcare (111), Medline (12), Cinahl (31), Scopus (7671), Informit (202), Google Scholar (200) and Google (200). In the screening process, 135 duplicate articles were removed and the remaining 8292 articles were screened on the basis of their abstract/title; 8249 articles were excluded. Most were excluded as they did not describe a pharmacy-delivered intervention with specific mention of ear health or were studies of a drug trial or drugs with ototoxic effects. The remaining 43 full-text articles were assessed for eligibility, with 32 full-text articles not meeting the inclusion criteria. These articles were excluded. The 11 remaining articles were deemed appropriate for inclusion in this scoping review.

### Identification and summary of included articles

The 11 included articles are summarised in Table 1. The articles included were conducted in Australia (*n* = 4), England (*n* = 4), United States of America (*n* = 2) and Brazil (*n* = 1). All articles were published since 2009, with two published after 2016. The ear health interventions identified included hearing screening (*n* = 3), otoscopy pilot studies (*n* = 2), audiometry services (*n* = 1), specific education for undergraduate pharmacy students (*n* = 2) and a pharmacy-based clinic (*n* = 3). The two otoscopy pilot studies described the same intervention that was reported by two different authors, with different perspectives, in two different professional practice journals and are thus reported separately in Table 2 (Independent Community Pharmacist, 2016; Weinbren, 2016).

**Table 2.** Inclusion and exclusion criteria

Criteria	Inclusion	Exclusion
Population	All populations including adults and children, pharmacy students, pharmacy interns and pharmacists	–
Intervention	An ear health intervention with a connection to community pharmacy.	Clinical drug trials
		Articles without a connection to community pharmacy practice
Outcome	Patient outcomes, health service outcomes, community pharmacy outcomes, patient and carer satisfaction, pharmacist and consumer perspective.	–
Comparison	No comparison	–
Time	All date ranges	–

The articles represented only two hierarchies of evidence, which were Level 3 – Promising Practices (*n* = 4) and Level 4 – Emerging Practices (*n* = 7). The Level 3 articles included a retrospective cohort study (*n* = 1), cross-sectional descriptive studies (*n* = 2) and a cohort study (*n* = 1). The Level 4 articles were all editorial articles including opinion letters (*n* = 1), service advertisements (*n* = 1) and articles from professional pharmacy practice magazines (*n* = 5). These Level 4 articles, although lacking methodological rigor, still provided insight into ear health interventions and thus were included in the review. None of the articles were conducted with Indigenous populations.

The *Deadly Ears Deadly Kids Deadly Communities Framework* guided the analysis of the articles (Durham *et al.*, 2015). None of the articles included Key Action Area 1 – *Prevention* or 6 – *Information and Knowledge*. Key Action Areas 2 – *Screening, Surveillance and Diagnosis* (*n* = 7), 3 – *Treatment and Support* (*n* = 8) and 5 – *Workforce Development* (*n* = 6) were most commonly linked to the articles. Key Action Area 4 – *Partnerships* could also be related to four articles.

#### Effectiveness and sustainability

Effectiveness and sustainability were the least commonly described elements of the interventions included in this review. In a North American study by Hall *et al.*, 408 patients were seen by a pharmacist providing an ear nose and throat service (32% OM cases). When participants were contacted 5 days later, 85% reported that they had not seen another health professional for their complaint, and none had attended a hospital emergency department providing some indication that the pharmacy-based service was effectively managing OM (Hall *et al.*, 2019). It is important to note that pharmacists were able to prescribe antibiotics in this study (Hall *et al.*, 2019). In this study, only 4.4% participants (*n* = 408) identified as Black, Asian or Minority Ethnic groups (Hall *et al.*, 2019). Another study with a focus on hearing loss, trained pharmacy students to counsel patients with a hearing impairment (Pisano & Miller, 2018). There was strong agreement from the students that the training program had enhanced their understanding of hearing loss (89%, *n* = 82) and improved their ability to identify and repair communication breakdowns (78%, *n* = 72). This study purely represented student’s perceptions post training, and no assessment of their skill was undertaken. Sustainability was not formally evaluated in any of the included articles; however, three articles described the inability to attract continuous funding and the importance of undertaking feasibility studies and economic evaluations for future models (Duncan *et al.*, 2016; Independent Community Pharmacist, 2016; Weinbren, 2016; Hall *et al.*, 2019). In addition, articles describing audiologist services being provided by external providers within communities pharmacies were described as continuing services (The Australian Journal of Pharmacy and The Australian Journal, 2014; Australian Pharmacist, 2016; Post Script, 2016; Warnbro Pharmacy, 2020).

#### Enablers and barriers

Enablers identified in the articles include positive outcomes, funding availability, consumer access to community pharmacy, cost savings for consumers and improved connection of pathways to health providers. A study of an ear, nose, throat and eye pharmacist service, which included prescribing medicines, reported a > 97% patient satisfaction rate, agreement that pharmacies are appropriate places to provide extended care services, agreement to use the service again and recommend it to others (Hall *et al.*, 2019). This service was funded by the North West Midlands Urgent and Emergency Care Network including training and supply of equipment (Hall *et al.*, 2019). Medicines recommended for the patients were purchased either by the patient or funded by the Pharmacy First Common Ailments Service (Hall *et al.*, 2019). In addition, top up funding was provided by the Local Professioanal Network for Pharmacy (Hall *et al.*, 2019). In the United Kingdom, a scheme has been implemented to allow pharmacists to manage minor ailments, including prescribing medicines for ear complaints, which has seen almost 20 000 pharmacist consultations occur over three years with an estimated 12 300 general practitioner (GP) appointments being freed up during this period (Lakhani & Eynon-Lewis, 2009).

Other reported enablers across all articles were pharmacist accessibility whereby pharmacies are described as easily accessible by most populations, the frequency of which people visit a community pharmacy, acceptability of the service by patients, saving GP time, reduced cost to the patients compared to GP visits, improved referral to hearing experts, stronger partnerships with hearing health professionals and the local community and a reduced burden on health systems (The Australian Journal of Pharmacy, 2014; Australian Pharmacist, 2016; Independent Community Pharmacist, 2016; Post Script, 2016; Weinbren, 2016).

Funding and consequently sustainability was also identified as a barrier with difficulty attracting funding to support ear health interventions reported (Weinbren, 2016). In particular, consideration of cost implications for training and equipment was described as a barrier to be considered for services that were to be developed at a national level (Hall *et al.*, 2019). Consequently, most of the interventions have been conducted as pilots, and no long-term financial investment has been secured to support the interventions to continue (Weinbren, 2016). Although there are limited funding models to support pharmacy-based ear health interventions, is has been determined that retail clinics are a less-costly alternative to GP settings for the treatment of episodes of acute OM (Duncan *et al.*, 2016). A cost reduction of between USD $30-$130 has been reported as well as confirmation that retail clinics were not duplicating GP services, in that patients visited one or the other service, not both (Duncan *et al.*, 2016).

Barriers identified through qualitative feedback provided in an opinion article written by doctors included: a lack of confidence in pharmacists ability to provide ear care interventions due to a lack of formal training; doctors belief that pharmacists are not equipped to make a differential diagnosis and that pharmacists would be working on a ‘best guess’ basis (Lakhani & Eynon-Lewis, 2009). In addition, there was concern that ear conditions may worsen because of a delay in patients seeking medical treatment, which may affect the outcomes for conditions including malignant otitis externa, herpes zoster infection, perichondritis, foreign body in the ear, referred pain from a tumour or temporal arteritis (Lakhani & Eynon-Lewis, 2009).

## Discussion

There is an urgent need for action to prevent and manage ear diseases and hearing impairment in Indigenous populations (Durham *et al.*, 2015). Indigenous populations have some of the highest rates of middle ear disease globally, which has led to significant financial investment and human resource application to address this stark inequity in hearing health. However prior investment has not produced a national data set or a national governance framework. It is not effectively reached remote locations, integrated telehealth or considered the community pharmacists role in ear health (Macquarie University, 2019). This highlights an opportunity to consider how community pharmacists can contribute to improving ear health in rural and remote communities.

### Key action area 1 – prevention

The prevention strategy encompasses stakeholder education, healthy lifestyle behaviours and improved public and environmental health (Durham *et al.*, 2015). None of the articles included in this scoping review described an intervention that included preventative strategies. This finding is of great importance given the frequency which patients visit their community pharmacy, an estimated fourteen times per year (The Pharmacy Guild of Australia, 2018). Pharmacies are well placed to engage in health promotion and the promotion of healthy lifestyles is described as one of the five core roles of a pharmacist. Extended opening hours and no need for appointments makes pharmacists a highly accessible health provider. However, a review of pharmacists’ involvement in public health found that pharmacists have low confidence in providing public health services, with reported barriers being limited time, inadequate counselling space, a lack of consumer demand and potential negative consumer reaction. Even so, consumers viewed pharmacists as appropriate providers of public health with high levels of satisfaction for those who had experienced a pharmaceutical public health service (Eades *et al.*, 2011). This highlights the opportunity for pharmacists to provide a greater role in health promotion and to discuss prevention strategies for ear disease with their patients; however, work is needed to unpack this potential.

### Key action areas 2 – screening, surveillance and diagnosis

Screening, surveillance and diagnosis to effectively manage ear conditions using a standardised and systematic approach is a major objective of the *Deadly Ears Deadly Kids Deadly Communities Framework* (Durham *et al.*, 2015). Working within 5700 community pharmacies across Australia are highly trained medication experts with knowledge and skills in health promotion (The Pharmacy Guild of Australia, 2018). This frequent and accessible health professional and patient connection provides an opportunity for pharmacists to engage people in healthcare and improve health outcomes.

In the study by Hall *et al.*, less than 5% of participants identified as Black, Asian or a Minority Ethnic groups. This highlights an important gap in that the service described by the study was not able to target the highest risk population group, limiting the generalisability of this study to an Australian Indigenous context. This finding is important to consider when developing future models to ensure consideration of how to ensure Indigenous Australians, and particularly children are included in the service delivery of innovative ear health interventions. Currently, community pharmacies are well recognised as easily accessible in rural communities; however, remote communities may be located some distance from a pharmacy and thus accessibility for those most isolated and most vulnerable may still present a challenge (The Pharmacy Guild of Australia, 2018).

### Key action area 3 – treatment and support

No article included in this review made specific mention on how they were ensuring the service was providing culturally appropriate treatment and support for patients, families and communities for ear health. It is known that providing culturally safe health care can contribute to improved health among Indigenous people. Culturally safe care is characterised by a genuine partnership between patients and healthcare providers in which: power is shared; the life experiences, views and beliefs, especially cultural beliefs are respected and Indigenous histories and social impacts are acknowledged. It is known that some Indigenous people feel culturally unsafe when using mainstream health services and this could include accessing pharmacies, which results in a reluctance to seek care (Gadsden *et al.*, 2019). Culturally safe practice should underpin all health professionals practice, and the Pharmaceutical Society of Australia has provided a guide to assist pharmacists to provide high-quality care for Indigenous populations. The guide has a focus on building relationships, understanding local protocols, communication and the provision of appropriate pharmacy services (Pharmaceutical Society of Australia, 2014). The importance of ensuring any newly developed pharmacy models are provided in the most culturally appropriate way is essential to ensure the most at risk population groups can comfortably access the services.

### Key action area 4 – partnerships

Pharmacists are one of the most trusted health professionals (The Pharmacy Guild of Australia, 2018). Public opinion surveys report that 95% of patients are satisfied with their community pharmacy (The Pharmacy Guild of Australia, 2018). This scoping review identified four publications that described this connection between community pharmacists and their local communities as being a major enabler for ear health interventions (Australian Pharmacist, 2016; Independent Community Pharmacist, 2016; Weinbren, 2016; Hall *et al.*, 2019). The opportunity for pharmacists to engage community members in ear health services both within community pharmacies and through referral to hearing specialists is an efficient method to improve partnerships and consequently improve ear health outcomes. Ensuring pharmacy-based ear interventions are linked to existing service providers will reduce the risk of fragmented patient care and improve community engagement with all services (Durham *et al.*, 2015).

### Key action area 5 – workforce development

In Brazil there is national legislation that requires all university health professional programs to include sign language as an elective course, except for speech pathology, for which the course is mandatory. It has been found that for pharmacy courses in Brazil, only half of the university curriculums meet the requirement and offer a sign language course. This is interesting as even in countries where there is legislation to include training specifically to target hearing impaired consumers in university courses, this is not occurring. For Australia there is no requirement to offer any training for university students to work with people who are hearing impaired and consequently it does not occur formally. It is clear that students learning sign language or a cultural language relevant to their communities for specific health- and pharmacy-related terminology would enhance patient care and medication safety (Souza Anacleto de Araújo *et al.*, 2019).

Three articles reported pharmacists receiving additional training to use an otoscope to examine an ear (Duncan *et al.*, 2016, Independent Community Pharmacist, 2016; Weinbren, 2016; Hall *et al.*, 2019). This was the only report of pharmacists undertaking any additional training to deliver ear health services. This is of importance as concerns about pharmacists skills and training to provide expanded services have been raised by other health professionals (Taylor *et al.*, 2019; Taylor *et al.*, 2020). It is important that when developing any new intervention that the providers are appropriately trained to provide the service to ensure that vulnerable patient groups are not receiving sub-optimal care.

### Key action area 6 – information and knowledge

The importance of maintaining accurate data and information by developing standard protocols for the collation and reporting of accurate ear and hearing health data is highlighted in this key action area. No articles found in this review described the integration of the pharmacy data into a larger health database. This is an important consideration as concern for pharmacy services to contribute to fragmented patient care has been identified as a risk to expanded pharmacy practice and improved integration will reduce this risk and improve patient care (Taylor *et al.*, 2019, Taylor *et al.*, 2020).

Overall, there are only a small number of ear health interventions by pharmacist being conducted (Duncan *et al.*, 2016; Independent Community Pharmacist, 2016; Weinbren, 2016; Hall *et al.*, 2019). Evidence demonstrating rigorous evaluation of ear health models to demonstrate effectiveness or the ability of the models to be sustained in the future is not found in the literature. There were no randomised controlled studies or longitudinal studies found in this review and no studies that demonstrated a significant level of intervention effectiveness or long-term sustainability. This is an important consideration for the development of innovative models of pharmacist-led ear health interventions. Ensuring appropriate evaluation of both effectiveness and sustainability is needed to be incorporated into the design of interventions to ensure that they are able to be provided past trial phases into future practice to improve ear health outcomes.

## Limitations

The study is limited in the search strategy whereby articles that may include an ear intervention as part of a larger intervention that did not describe ear/s in the title, abstract or keywords would not have been found.

## Conclusions

There is very little evidence to support the wide adoption of a pharmacy implemented ear health model of care. Regardless, closing the gap between Indigenous and non-Indigenous Australians in hearing health is of high social and economic value, and solutions to the barriers of accessibility and engagement are urgently needed. A community pharmacist is accessible, highly trained and is thus well placed to pilot innovative ear health interventions, particularly in rural, remote and Indigenous communities, where shortages of other health professionals are acute. Future study to explore the design, development and trial of ear health models for community pharmacy practice will require community and stakeholder consultation and support, a pharmacy training program and funding to ensure sustainability.

## Statements

None of this material has been published previously, is under consideration, or has been accepted for publication elsewhere.

No conflict of interests to declare.

AC is supported by an NHMRC-funded Fellowship ‘Improving Health Outcomes in the Tropical North: A multidisciplinary collaboration (Hot North)’, grant identification number 1 131 932’. The content of this publication is solely the responsibility of the authors and does not reflect the views of the NHMRC.

All persons listed as authors have read and given approval for the submission of the manuscript.

The head of the department in which the work was carried out has given permission for the manuscript to be published.
